# The Impact Mechanism of Green Credit Policy on the Sustainability Performance of Heavily Polluting Enterprises—Based on the Perspectives of Technological Innovation Level and Credit Resource Allocation

**DOI:** 10.3390/ijerph192114518

**Published:** 2022-11-05

**Authors:** Xiaowei Ding, Ruxu Jing, Kaikun Wu, Maria V. Petrovskaya, Zhikun Li, Alina Steblyanskaya, Lyu Ye, Xiaotong Wang, Vasiliy M. Makarov

**Affiliations:** 1 Faculty of Economics, RUDN University, 117198 Moscow, Russia; xiaoweiding06796@gmail.com (X.D.); petrovskaya-mv@rudn.ru (M.V.P.); 2 Institute of Economics, Moscow State University, 119991 Moscow, Russia; ruxujing525@gmail.com; 3 Institute of Economics and Management, Lviv Polytechnic National University, 999146 Lviv, Ukraine; juziwukaikun@163.com; 4 Institute of Asian and African Studies, Moscow State University, 119991 Moscow, Russia; lizhikun756@gmail.com; 5 School of Economics and Management, Harbin Engineering University, Harbin 150009, China; alina_steblyanskaya@hrbeu.edu.cn; 6 Institute of Industrial Management, Economics and Trade, Peter the Great St. Petersburg Polytechnic University, 195251 Saint Petersburg, Russia; vmmak51@mail.ru

**Keywords:** green credit policy, credit resource allocation, technological innovation level, heavily polluting enterprises, corporate sustainability performance

## Abstract

Green credit policy (GCP), as one of the key financial instruments to achieve ’carbon peaking’ and ‘carbon neutrality’ targets, provides capital support for the green development of enterprises. This paper explores the impact mechanism of GCP on the sustainability performance of heavily polluting enterprises (HPEs) from the perspectives of technological innovation level (TIL) and credit resource allocation (CRA), using panel data for Chinese A-share listed manufacturing companies from 2010 to 2015 to construct a propensity score matching and differences-in-differences (PSM-DID) model. We find that GCP has a causal effect on corporate sustainability performance (CSP). Although GCP significantly improves CSP, there is no long-term effect. Heterogeneity analysis shows that the relationship between GCP and CSP is only significant in non-state-owned enterprises and in eastern and low-market-concentration enterprises. Mechanism tests indicate that GCP stimulates HPEs to invest more in technological innovation and thereby improves CSP through the innovation compensation effect; the credit constraint and information transfer effects caused by GCP reduce the credit resources available to HPEs but have a significant forced effect on CSP. This paper enriches the study of the economic consequences of GCP and provides implications for stakeholders to improve the green financial system and achieve green transformation of HPEs.

## 1. Introduction

Green and low-carbon production and lifestyle are the common and beautiful pursuits of all mankind. How to balance the relationship between economic development and environmental protection has become an urgent and realistic issue for countries around the world. With the rapid industrialization and urbanization of China, environmental pollution has become a non-negligible problem in the process of high-speed economic development [1]. Ecological civilization construction requires the coordinated development of the economy and environment, and environmental governance cannot be achieved without financial resource support. Green finance has emerged as a link between the environmental and financial sectors. As an important policy tool for modern environmental governance, green finance has the dual characteristics of financial resource allocation and environmental regulation and is a useful complement to traditional environmental regulation policies [2]. Green credit is an important green financial policy that requires financial institutions to take pollution control and environmental protection as an essential basis for credit decisions and to guide the green transformation of industrial structures through credit resource allocation (CRA) [3]. In 2012, the China Banking Regulatory Commission (CBRC) released the ’Green Credit Guidelines’, which is an innovative practical policy for the central government to use financial regulation tools in environmental governance.

At present, research on green credit policy mainly focuses on implementation risks and implementation effects. Zhao and Chen [4] analyzed the risks and uncertainties of green credit and believe that government intervention, green technology innovation and regulator behavior are macro factors that influence green credit risk. The economic consequences of policy implementation are reflected at the macro and micro levels. At the macro level, the essence of green credit policy is environmental policy. Its implementation not only promotes the efficiency of the regional green economy but also generates spatial spillover effects, which contribute to the green economy development of neighboring regions and share the environmental and policy dividends between local governments and enterprises [5,6]. The fundamental purpose of the GCP is to reduce corporate pollution emissions. At the starting stage of the policy implementation, the environmental quality has not been significantly improved, and the policy effect has not been revealed; with the continuous improvement of the green financial system, the relationship between GCP and air quality shows a significant positive effect [7]. In the context of widening green capital shortages, green credit policy can guide the flow of capital within the financial system from heavily polluting industries to green industries, which significantly improves the efficiency of energy utilization and environmental governance capacity [8,9,10]. Meanwhile, the policy improves environmental quality and green total factor productivity by stimulating innovation potential and upgrading the industrial structure [11].

At the micro level, green credit policy strengthens the supervisory function of financial institutions towards users of funds and enhances their own environmental and financial performance, which significantly promotes their high-quality sustainable development [12]. However, the implementation of green credit policy could not significantly reduce the non-performing loan ratio and has a negative impact on banks’ return on equity [13]. As a major player in the market economy, enterprises are not only the main producers of environmental pollution but also the main contributors to environmental governance. The unbalanced allocation of credit resources objectively promotes the expansion of polluting investments, leading to increased environmental pollution and higher governance costs. Therefore, there are objective conditions for the implementation of green credit policy (GCP), but the policy is not entirely conducive to corporate development. The green credit policy may inhibit enterprise growth. Through the differential credit constraint approaches to allocating resources, the scale of debt financing for heavily polluting enterprises (HPEs) is significantly reduced [14]. At the same time, it is undeniable that the implementation of GCP has an important impact on enterprise investment efficiency. Zhang et al. [15] believe that the implementation of GCP limits the efficiency of corporate domestic investment, and the crowding-out effect of high debt financing costs inhibits the green innovation activities of HPEs. In contrast, Xing et al. [16] and Zhang et al. [17] find that GCP improves the efficiency of overseas investment by Chinese enterprises, and that the policy effect is more significant for SOEs as well as for companies in regions with low environmental regulations. In addition, increased financial constraints and reduced information transparency lead to green credit policies significantly increasing the risk of stock price collapse for HPEs [18]. GCP may promote the green transformation of enterprises. The punishment effect of financing constraints forces HPEs to change their operation methods and achieve green upgrading of the industrial structure. GCP through favorable lending rates and green innovation compensation generates higher incentive effects than the punishment effects of financing constraints [19,20], and differentiation competitive advantage and first-mover advantage accelerate the green transformation of enterprises [21,22]. As an essential component of green supply chain practices, GCP plays an important role in corporate sustainability. Kang et al. [23] find that the implementation of GCP can create added value by motivating manufacturers to support their suppliers’ pollution reductions. In the context of GCP implementation, manufacturers have a higher capacity to reduce emissions if emissions of pollutants are reduced by their suppliers.

Above all, existing studies describe the financing constraint punishment effect and innovation compensation effect of GCP. Research on the economic consequences of GCP remains controversial due to differences in analytical methods and perspectives. In addition, sustainability performance is a composite measure of corporate financial and environmental performance, which reflects whether a company is making the best use of relevant resources for value creation and environmental protection. There is still a shortage of studies from this perspective. Considering that HPEs are the primary source of environmental pollution, the GCP aims to support green economy development by limiting the flow of funds to polluting areas. Using the ‘Green Credit Guidelines’ as a quasi-natural experiment to analyze the impact of GCP on corporate sustainability performance (CSP) is the best way to objectively reflect the effects of GCP. Therefore, the purpose of this paper is to explore the impact mechanism of GCP on the sustainability performance of HPEs through panel data of Chinese A-share listed companies from 2010–2015. 

The main contributions of this paper are as follows: (1) The propensity score matching (PSM) method is introduced to filter the covariates, which indicates that the standardized mean difference (SMD) values of all covariates are less than 0.2. This method significantly reduces the sample selection bias and improves the reliability of the results. (2) Verifying not only that the ‘Green Credit Guidelines’ policy significantly contributes to the improvement of the technological innovation level (TIL), but also that there is a significant positive relationship between GCP and CSP, these findings enrich the applicability of the Porter effect in the Chinese manufacturing industry. (3) This paper shows that there is a causal effect between the ‘Green Credit Guidelines’ policy and the sustainability performance of HPEs, but that their positive relationship does not have a long-term effect, and the policy effect gradually diminishes as time increases. (4) The reliability of the baseline regressions is verified using four methods: replacing the dependent variable, counterfactual test, shortening the time window and introducing other policies. The innovations of this paper are that: (1) We do not study the impact of GCP on financial performance or environmental performance, but rather, by using the entropy method to construct a composite index of CSP from financial and environmental social responsibility dimensions, we verify that GCP can significantly improve CSP, thereby enriching the theoretical study of the relationship between GCP and CSP. (2) Control variables are selected from the firm and regional aspects to make the regression results more convincing. (3) The heterogeneity of the relationship between GCP and CSP from the internal characteristics of enterprises and the external environment is verified; the impact of GCP on CSP is more significant among non-state-owned enterprises (SOEs), eastern firms, firms with low market concentration and high green innovation firms. (4) The impact mechanism of GCP on the sustainability performance of HPEs from the perspectives of TIL and CRA is explored; GCP can significantly improve the sustainability performance of HPEs through credit financing and technological innovation. This study enriches the theory of green credit and provides insights for sustainable corporate development and government green financial system improvement.

Section 2 is a theoretical analysis and hypothesis formulation concerning GCP, TIL, CRA, CSP and mediating effects. Section 3 represents the materials and method. Section 4 is the results. Section 5 concerns extended research. Section 6 includes discussion and Section 7 provides a conclusion. 

## 2. Theoretical Analysis and Hypothesis Formulation

### 2.1. The Relationship between GCP and CSP

GCP can increase the level of external financing constraints faced by enterprises, which is essentially the impact of environmental regulation on the behavior of HPEs. GCP combines administrative penalties to both limit the financing capacity of HPEs and increase their environmental costs to stimulate enterprises to undertake environmental governance and green transformation [24]. GCP has a significant impact on all aspects of business such as total factor productivity, environmental responsibility, carbon efficiency, sustainability performance, etc. Green technical change and green efficiency change are used as indicators for measuring green total factor productivity. Liu et al. [25] find that GCP has a significant negative effect on green technical change but has a significant positive relationship with green efficiency change. However, the positive effect is greater than the negative effect, and the implementation of GCP significantly increases the green total factor productivity of HPEs. The implementation of GCP makes the financial department adopt environmental factors as one of the conditions for granting credit to enterprises, thus injecting more credit resources into green projects. However, due to the information asymmetry in the capital market, the actual green level of enterprises is difficult to observe, so enterprises must send signals to financial institutions through their own green behavior to ensure an advantage in their credit activities. One of the most accessible ways to do this is to take on environmental social responsibility or to disclose environmental information to send a ‘green signal’ to financial institutions in order to obtain more credit resources [26]. Zou and Wang [27] use the SBM super-efficiency model to measure the carbon emission efficiency of China’s provinces and find that green credit policies significantly improve carbon emission efficiency through the improvement of green technology and the optimization of factor structure.

CSP is a composite indicator that evaluates corporate financial, environmental and social responsibility performance. Jiang et al. [28] empirically verify that GCP can significantly improve CSP using the PSM-DID model. In addition, Aller et al. [29] take a study of listed new energy companies in China and show that GCP can increase the value of new energy companies by alleviating financing constraints and strengthening external regulation, and that this positive impact can be long lasting. GCP also has a positive impact on the sustainability performance of financial institutions. Zhang and Zhou [30] show that GCP makes a significant contribution to the high-quality sustainable development performance of banks. Xi et al. [31] use the green credit ratio and green reputation as indicators to measure the quality of GCP implementation in listed banks and verify that the quality of GCP implementation is significantly and positively correlated with the financial performance of listed banks.

To summarize the above studies, we believe that GCP has the characteristics of the command–control type of environmental regulation, forcing HPEs to carry out green upgrading of their industrial structure, and thereby improving environmental performance [32]. It also has the characteristics of a market-incentive type of environmental regulation, which stimulates enterprises to improve their financial performance by investing in technological innovation, developing cleaner production, and dominating the market through differentiation competitive advantage and first-mover advantage. Thus, the following hypotheses are proposed:

**Hypothesis 1** **(H1).** *The GCP significantly enhances CSP.*

### 2.2. Heterogeneity in the Relationship between GCP and CSP

From the supply-side perspective of financial resources, GCP forces HPEs to develop green projects through CRA, thereby achieving the green upgrading of industrial structure. However, based on the demand-side perspective, the sensitivity of GCP may vary among enterprises with different characteristics, leading to heterogeneity in economic consequences.

China’s special political system determines the dominance of SOEs in the national economy. For a long time, under a highly centralized financial system dominated by large state-owned banks, there has been the phenomenon of a ‘political-pecking order’ in credit resource allocation in China [33]. External financing constraints are the most important factor that inhibits the development of real enterprises. SOEs have natural political connections and government credit endorsement [34], making it easier to access credit resources, tax incentives and government subsidies. The bank loan is one of the most important external financing methods in China, and private financing is not well developed due to imperfect policies and regulations. Therefore, non-SOEs face higher credit constraints. Only if a non-SOE actively implements environmental protection policies, assumes environmental responsibility, improves investment in technological innovation and takes the initiative to improve the quality of environmental information disclosure, will it thereby acquire more credit resources by signaling green transformation to financial institutions. The impact of GCP on non-SOEs is greater than that on SOEs [35,36]. Based on the above, this paper proposes the following hypothesis:

**Hypothesis 2a** **(H2a).** *The GCP has a more significant impact on the sustainability performance of non-SOEs than that of SOEs.*

Regional coordinative development policy implemented by the central government reduces the economic gap among regions to a certain extent but does not fundamentally change the local economic development model and the political environment. The green credit policy has a positive impact on the upgrading of China’s industrial structure and significantly contributes to green and advanced industrial structure [37]. Technological innovation is an important factor in promoting regional economic development. Driven by technological progress, factors of production and resources are transferred from low-productivity sectors to high-productivity sectors, which ultimately promotes the optimization and upgrading of industrial structures. However, China’s regions are at different stages of economic development, and non-eastern regions are lagging behind in terms of economic development and are unable to attract sufficient capital and introduce advanced technology to promote industrial structure upgrading [38]. Healthy development of the financial market can create a good financial ecological environment for enterprises and effectively relieve their financing pressures. In the eastern region, the level of financial development is high and the capital market is active. As a result, financial institutions can obtain more detailed information about borrowing enterprises and can effectively evaluate the quality of enterprises’ investment projects with less information asymmetry problems. In contrast, in non-eastern regions, the level of financial development is relatively low, and the problem of information asymmetry is serious, which increases the approval and supervision costs of financial institutions [39]. The eastern regions have a higher level of openness to the outside world and economic development, a more robust political system and more transparent information on environmental protection [40]. Compared to the eastern regions, the non-eastern regions are underdeveloped and still carry out the ‘development before governance’ production model [41]. The lack of supervision by public opinion and the outdated structure of the political system result in more corruption. Moreover, to help local economic development, the central government provides more financial subsidies and lower tax policies for enterprises. There are significant differences between regions in terms of industrial structure, technology level and government environmental regulation. Therefore, after the implementation of the green credit policy, although the financing environment faced by heavily polluting enterprises for green innovation activities is more severe, the implementation of GCP has insignificant effects on non-eastern regional enterprises. Therefore, we propose the following hypothesis:

**Hypothesis 2b** **(H2b).** *The GCP contributes more significantly to the sustainability performance of eastern enterprises than that of non-eastern enterprises.*

Industry characteristics determine that there is significant variation in the intensity of competition among enterprises. GCP as an environmental regulation can significantly reduce the credit financing capacity of HPEs, but oligopolistic enterprises that are at high market concentration have an absolute advantage in the market [42]. As a result, these enterprises are not sensitive to GCP. However, competitive enterprises at low market concentration face greater competitive pressures, and the compliance cost created by GCP increases financing constraint strength and requires higher environmental information disclosure quality. High external pressures force them to improve their green innovation level and to rely on competitive differentiation advantage and first-mover advantage to rapidly gain market share and thereby improve their CSP. Therefore, this paper proposes the following hypothesis:

**Hypothesis 2c** **(H2c).** *The positive impact of GCP on the sustainability performance of enterprises with low market concentration is more significant than that of enterprises with high market concentration.*

### 2.3. The Relationship between GCP, TIL and CSP

Green credit is an environmental economic policy in which financial institutions consider corporate environmental performance to be a requirement for credit approval. From the perspective of the compliance cost effect, the implementation of GCP can increase the production cost and pollution control cost of enterprises, which has a crowding-out effect on R&D investment activities and reduces the productivity of enterprises. Li et al. [43] propose the opposite conclusion by using a Super-SBM model that includes unintended outputs to verify that GCP significantly and positively affects the green innovation efficiency of HPEs, but with a lagged effect. Further research finds that green technological innovation through increased R&D investment leads to the green transformation of HPEs. Therefore, based on the innovation compensation effect of the Porter hypothesis, when the external pressure generated by green credit affects the survival of enterprises, HPEs generate a huge market demand for environmental technology, which stimulates them to increase their R&D investment to achieve technological progress and significantly increases the output of technological innovation [44,45].

We believe that innovation compensation effects can offset compliance costs to generate net benefits, thereby improving production management efficiency and corporate competitiveness. Following the implementation of GCP, green innovation can improve the financial, environmental and social performance of companies, while ‘greenwashing’ can harm the performance of companies [46]. Based on the above, this paper proposes the following hypothesis.

**Hypothesis 3** **(H3).** *GCP can significantly increase TIL, thereby positively affecting CSP.*

### 2.4. The Relationship between GCP, CRA and CSP

Loans distributed by Chinese financial institutions accounted for 63.6% of social financing in 2021, and the main source of debt financing for China’s real economy is bank borrowing. Commercial banks strictly regulate the audit system for green credit; in addition to considering the financial indicators of the lending enterprises, they also pay attention to corporate environmental performance. The implementation of GCP can increase financing difficulties for companies in the short term, reduce external credit resources and generate credit constraint effects. Zhang et al. [47] find that GCP has an incentive effect on the short-term financing behavior of HPEs but a punishment effect in the long term, significantly inhibiting corporate investment behavior and improving environmental performance. The same conclusion is also reached by Wang et al. [48]. Although GCP inhibits capital investment by HPEs, there is a forcing effect which pushes enterprises to upgrade their industrial structure to improve their total factor productivity, thereby achieving a win–win outcome for economic development and environmental protection. Based on the above, we put forward the following hypothesis:

**Hypothesis 4** **(H4).** *The GCP has a positive impact on CSP by adjusting the CRA of HPEs.*

**Figure 1:**
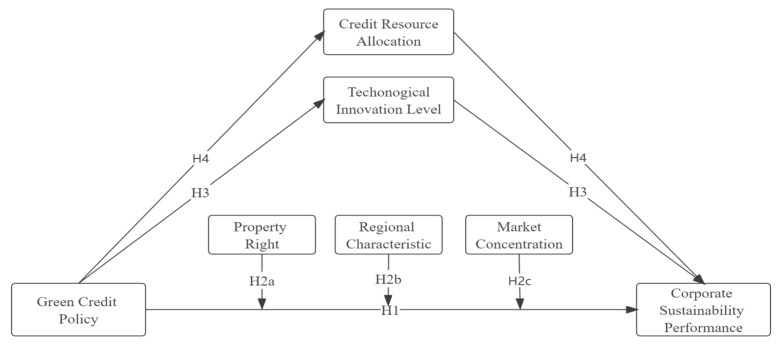
Conceptual model.

We develop a conceptual model in this paper based on the above hypotheses, as shown in Figure 1.

## 3. Materials and Method

### 3.1. Sample Selection and Data Sources

The definition of heavily polluting enterprises is based on the ‘Guidelines on Environmental Information Disclosure for Listed Companies’ published by the State Ministry of Environmental Protection in 2010, and we collate the heavily polluting industries using the industry classifications of the ‘China Securities Regulatory Commission (2012 edition)’. This paper takes Chinese A-share listed manufacturing enterprises from 2010–2015 as the sample and refers to studies by Yang and Niu [49] and Liu and Shao [50] to classify 15 heavily polluting industries, such as coal mining, as the treatment group and 16 non-heavily polluting industries, such as food manufacturing, as the control group. The details of the industry attribution groups are shown in Table A1 (Appendix A). To ensure data validity, samples with missing data, extreme values and those labelled ‘ST’ and ‘*ST’ are excluded. Of these, special treatment is applied to trading in the shares of listed companies with unusual financial conditions, and companies that have been ‘specially treated’ are referred to as ‘ST’; companies that have been ’specially treated’ and have suffered losses for three consecutive years and are at risk of delisting are referred to as ‘*ST’. Ultimately, we obtained 1224 valid observations for 205 companies, including 611 observations for the treatment group and 613 observations for the control group. The data used to measure financial performance are from the China National Research Data Service (CNRDS) platform, the data of environmental performance are from the Hexun.com database, the data of regional control variables are from the National Bureau of Statistics of China (NBS) and the China Market Index Database (CMI), the data for the corporate control variables are from the China Stock Market & Accounting Research Database (CSMAR), and the other data are from the WAND (Wind) database. The data were processed and analyzed using Python 3.8 and Integrated Development Environment (IDE) PyCharm 2021.2.2 software.

### 3.2. Variable Identification and Sample Description

#### 3.2.1. Dependent Variables

We refer to the study by Alexopoulos et al. [51], which divides CSP into two dimensions, financial performance and environmental performance, and calculates a composite index of sustainability performance using the entropy method. Based on the method of DuPont analysis, return on equity (ROE) is used as an indicator of corporate financial performance. Referring to the study by Jiang et al. [28], we use the corporate environmental responsibility (CER) score from the Hexun.com database as an indicator of corporate environmental performance. The CER score consists of five dimensions: environmental awareness, environmental management system certification, amount of environmental investment, number of types of emissions, and energy savings, which can better evaluate the ability of enterprises in emissions reduction.

#### 3.2.2. Independent Variables

We use the implementation of the ‘Green Credit Guidelines’ as a quasi-natural experiment, and $Treated_{i}\times Post_{t}$ is the interaction term used to represent the net effect of GCP on CSP, where $Treated_{i}$ is the policy target, $Treated_{i}=1$ means HPEs, and $\;Treated_{i}=0$ means non-heavily polluting enterprises (non-HPEs); $Post_{t}$ is the implementation time of policy, as China formally implemented the ‘Green Credit Guidelines’ policy on 1 January 2012, $Post_{t}=1$ indicates the time period after the policy implementation (including 2012), and $Post_{t}=0$ indicates the time period before the policy implementation.

#### 3.2.3. Mediating Variables

After the GCP was issued, commercial banks would use corporate environmental information as the basis for granting credit. Therefore, the GCP constrains corporate finance through the financing punishment effect. According to the strong version of Porter’s hypothesis, technological innovation is an important approach to achieve green transformation. Moreover, technological innovation activities are characterized by high risk and long terms. Only companies with sufficient, long-term available capital can satisfy the requirements of technological innovation activities. Therefore, we further explore the internal transmission effect between GCP and CSP by using total long- and short-term credit financing capacity, long-term credit financing capacity and technological innovation investment level as mediating variables.

#### 3.2.4. Control Variables

We select company size, financial leverage, growth, nature of ownership, percentage of independent directors and financial background of directors as corporate control variables. Moreover, as the green credit policy has different effects in different regions, we use the level of market development and the level of economic development as regional control variables. The specific variables involved in this paper and their descriptive statistics are presented in Table 1.

### 3.3. Research Method

**Figure 2:**
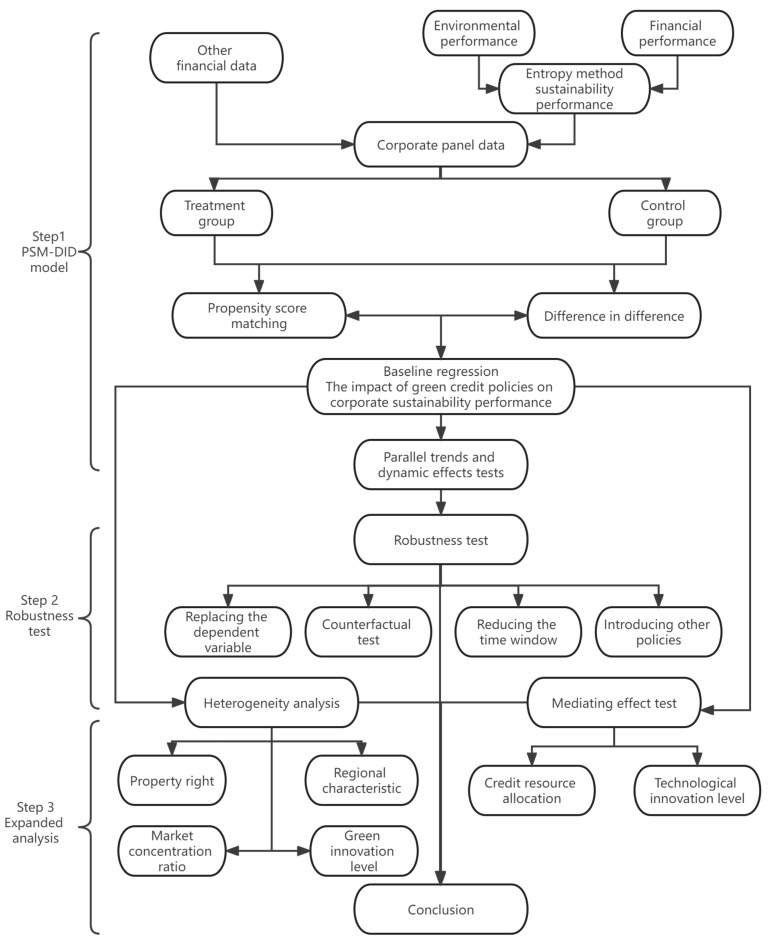
Structure of an integrated model for the impact of GCP on CSP.

In this paper, we propose an integrated model to explore the impact mechanism of GCP on CSP, as shown in Figure 2.

Step 1. The PSM-DID model: Financial performance and environmental performance are the most effective indicators of CSP. We use the entropy method to calculate a composite index of sustainability performance and compare panel data about enterprises with other financial information. The sample enterprises are classified according to the environmental attributes of the manufacturing industry, and HPEs are used as the treatment group and non-HPEs are used as the control group. Since classifying enterprises based on environmental attributes is prone to selection bias, we use PSM to screen valid covariates to make the conclusions of this paper more accurate. The impact of GCP on CSP is analyzed by the PSM-DID model and tested for parallel trends and dynamic effects.

Step 2. Robustness test: The omission of variables may lead to unreliable conclusions from the baseline regression. We use four methods, namely, replacing the dependent variable, conducting a counterfactual test, reducing the time window and introducing other policies, to verify the reliability of the baseline regression results.

Step 3. Extended research: To further explore the impact mechanism of GCP on CSP, we conduct empirical analyses from the perspectives of heterogeneity and mediating effects. Differences in the internal nature and external environment of enterprises may lead to heterogeneity in the relationship between GCP and CSP. Therefore, we test them in terms of four dimensions: nature of ownership, market concentration, green innovation level and regional characteristics. How does GCP affect CSP? This question is the focus of our research. Commercial banks are the main implementers of GCP, and Chinese enterprises’ sources of debt financing are mostly bank borrowing. The strong version of Porter’s hypothesis suggests that environmental regulations stimulate companies to invest more in technological innovation to gain a first-mover advantage. Therefore, we test whether there are mediating effects in the relationship between GCP and CSP from the perspective of CRA and TIL. Finally, we conclude the paper with meaningful recommendations for companies, government departments and other stakeholders.

## 4. Results

### 4.1. Propensity Score Matching and Analysis of Causal Effects

To improve the comparability between the treatment and control groups, we use PSM method to reduce sample selection bias. *Size*, *Lev*, *Growth*, *State*, *Idr*, *Fbd*, *Gdp* and *Mai* are selected as two groups of corporate characteristic variables. In the matching process, a Logit regression model is constructed with the policy target as the dependent variable, and the regression model is formulated as follows:

(1) $$Treated_{i}=\beta_{0}+\beta_{1}Size_{it}+\beta_{2}Lev_{it}+\beta_{3}Growth_{it}+\beta_{4}State_{it}+\beta_{5}Idr_{it}+\beta_{6}Fbd_{it}+\beta_{7}Gdp_{it}+\beta_{8}Mai_{it}+\varepsilon_{it\;\;\;\;\;}$$

where

i  is the firm; 

t is the year; 

α is the coefficient;

Treated is the target of policy implementation;

$\varepsilon_{i}^{t}$ is the residual.

**Figure 3:**
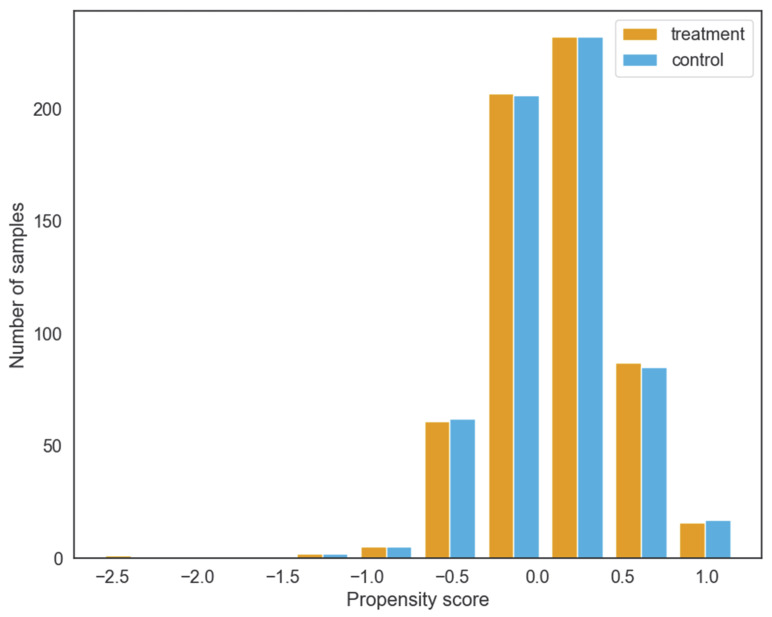
Propensity score distribution of treatment and control.

Since the treatment and control groups include companies from different industries, there can be some differences in the characteristics of the companies in the two groups, and these differences may affect the estimation results. In addition, the trends in the two groups may be different in the DID estimate before the implementation of GCP. Therefore, the preconditions for using the propensity score matching method are that the common support test and the balance test are satisfied to ensure that the matching effects of the samples meet the requirements. We carry out matching using the psmpy package developed by Kline and Luo [52]. In this paper, the nearest neighbors algorithm with matching with replacement is used; the caliper size is set to 0.05, and matching is performed based on the propensity score. The result of the common support test is shown in Figure 3. The propensity score distributions of the vast majority of samples in the treatment and control groups are very similar after matching, and the matching accuracy is guaranteed. It is necessary to point out that we set the caliper value based on the nearest neighbor matching (NNM) method, which effectively solves the problem that the method of NNM has difficulty guaranteeing the matching quality when the distribution of propensity scores between the treatment group and the control group is widely different. Furthermore, although the number of samples in the control group is decreased by using matching with replacement, the bias reduction improves the fitting ability of the algorithm and the overall matching quality [24,53].

This paper uses *t*-test and quantitative indicators to evaluate the effect of the balance test. The results of the *t*-test are shown in Table 2, where the *p*-values of all covariates are greater than 10%. This result indicates that after matching there is no significant difference between the two groups for any covariates. In the following, we use quantitative indicators to verify the findings of the *t*-test.

**Figure 4:**
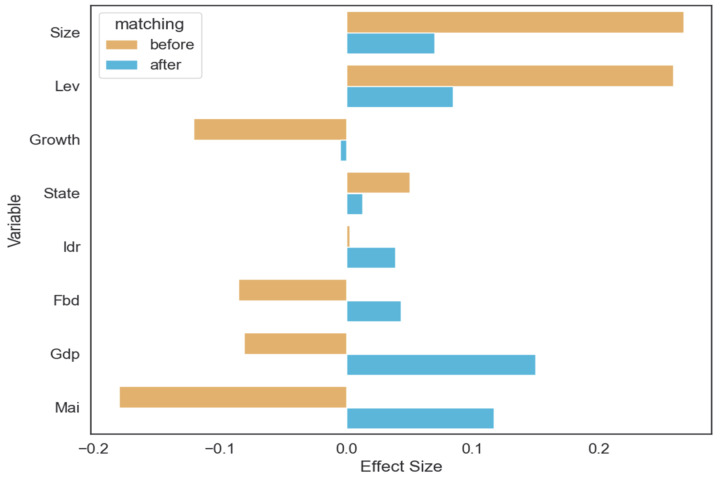
Standardized mean differences of covariates before and after matching.

This paper uses the SMD as a quantitative indicator to evaluate the quality of matching. Cohen [54] set a threshold for effect size: small ≤ 0.2, medium ≤ 0.5, large ≤ 0.8. After matching, the effect size imposed by variables should be smaller than that before matching, and the closer the effect size is to zero, the less dependent are the estimate results on this covariate. As shown in Figure 4, the SMD values of most covariates decreased significantly after matching. Moreover, the absolute values of SMD for all covariates are less than 0.2. In summary, all covariates pass the equilibrium test.

To assess whether there is a causal effect between the implementation of GCP and CSP, we estimate the average effect of treatment on the treated (ATT) by ordinary least squares (OLS) using a causal inference package. The results are shown in Table 3, where we find that ATT is 0.048 and significant at the 1% level. Therefore, there is a causal effect between GCP and CSP after matching.

### 4.2. Baseline Regression Results

To effectively estimate the net effect of the policy while addressing the sample selection bias, this paper constructs a PSM-DID model to explore the impact mechanism of GCP on CSP. After matching, valid observations are obtained for 186 companies, including 610 observations for the treatment group and 154 observations for the control group. The baseline model for PSM-DID in this paper is set as follows:

(2) $$Csp_{it}=\alpha_{0}+\alpha_{1}Treated_{i}\times Post_{t}+\alpha_{2}Controls_{it}+Fixed\;effects+\varepsilon_{it}$$

where

i is the firm;

t is the year;

α is the coefficient;

Controls is the set of control variables;

Fixed effects is the two-way fixed effects;

$\varepsilon_{it}$ is the residual.

Based on Equation (2), we use stepwise regression to estimate the effect of GCP on CSP with and without the introduction of control variables. In Table 4, model (1) shows that GCP is significantly and positively correlated with CSP at the 5% level when control variables are not introduced, and model (3) shows that GCP is still significantly and positively correlated with CSP at the 1% level when control variables are introduced. These results indicate that the implementation of GCP can significantly contribute to CSP. These findings fully support H1. The introduction of control variables significantly improves the fit of the model. In addition, *State* and *Fbd* have a significant positive impact on CSP.

### 4.3. Analysis of Parallel Trends and Dynamic Effects

Currently, there are two methods to test for parallel trends: (1) comparing the time trends of the means of the dependent variables in the treatment and control groups, and if the trends are the same, then there is a parallel trend in the two groups; and (2) adding the interaction term $Treated_{i}\times Post_{t}$ at each time point in the regression model, and if the coefficient of the interaction term before the implementation of the policy is not significant, then it indicates that there is a parallel trend. The second method is used for testing in this paper. We chose a time window of six years from 2010–2015, and the following dynamic model is constructed to capture the parallel trends between the treatment and control groups before the implementation of the policy and the dynamic effects of the policy on CSP after implementation.

(3) $$Csp_{it}=\beta_{0}+\sum \beta_{1}Treated_{i}\times Post_{t}+\beta_{2}Controls_{it}+Fixed\;effect+\varepsilon_{it}$$

where

i is the firm;

t is the year;

$\beta_{1\;}$ is the DID coefficient, which measures the dynamic effects of GCP before (period 2010–2011) and after (period 2012–2015) the policy was issued;

$\beta_{2}$ is the coefficient of the control variables;

Controls is the set of control variables; 

Fixed effects is the two-way fixed effects; 

$\varepsilon_{it}$ is the residual.

**Figure 5:**
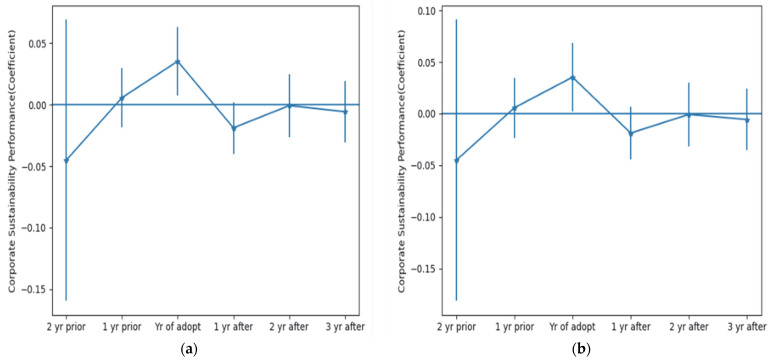
A multi-period DID model for testing parallel trends and dynamic effects on corporate sustainability performance. (**a**) 90% confidence interval; (**b**) 95% confidence interval.

The test results are shown in Figure 5. Before the implementation of the policy, Figure 5a,b show that the confidence intervals for the interaction terms from 2010–2011 both contain 0, meaning that the original hypothesis is accepted and the coefficient of the interaction term is not significant. Therefore, there is a parallel trend between the treatment and control groups after matching. The Green Credit Guidelines policy was formally implemented in 2012, and both Figure 5a,b show that the confidence interval of the interaction term $Treated_{i}\times Post_{12}$ does not contain 0 and the coefficient is significant. In addition, the confidence intervals for the years 2013 to 2015 interaction terms do contain 0 and the coefficients are insignificant. These findings indicate that there is no long-term effect of GCP on the sustainability performance of HPEs, with the effect of policy implementation diminishing over time. The main reason for this is that China’s green financial reform started late and did not have a complete package of measures in place when implementing the Green Credit Policy (GCP). In addition, despite the Chinese government’s efforts to create a favorable business environment, corruption occurs due to the high level of government–business nexus as a result of China’s special political system and inadequate media oversight. Local officials conspire with corporate managers to exempt companies from environmental penalties, or fail to disclose environmental information in the pursuit of political achievements [50]. Enterprises fraudulently obtain green credit resources from banks through ‘greenwashing’ behaviors. GCP may be implemented in a biased manner, reducing policy effect. The implementation of GCP has a negative impact on the financial performance of commercial banks, which may reduce the criteria for granting green credit. Furthermore, green credit policy (GCP) not only strengthens corporate financial constraints, but also reduces investment efficiency and the scale of debt financing, causing policy performance to be offset by corporate losses, resulting in insignificant long-term policy effects [55,56].

### 4.4. Robustness Test

The baseline regression shows that the implementation of GCP significantly enhances CSP. To verify the reliability of this finding, this paper uses four methods, including replacing the dependent variable, counterfactual, shortening the time window and introducing other policies for robustness testing. The results of the tests are shown in Table 5. In model (1), we replace ROE with return on assets (ROA) as a measure of financial performance and use the entropy of ROE and the natural logarithm of environmental performance as the new sustainability performance (Csp_new). The results show that GCP has a significant positive impact on CSP at the 1% level. Robustness tests for replacing the dependent variable are passed.

The counterfactual test means that GCP is chosen to be implemented at a dummy time, and since GCP is not truly implemented at that time, CSP is not affected by GCP. If the net effect coefficient is significant, then the baseline regression result is not valid; if the coefficient is not significant, then the counterfactual test is passed. The paper uses 2010 as the time of GCP implementation for the counterfactual test. Model (2) shows that the coefficient of $Treated_{i}\times Post_{10}$ is not significant after changing the time of implementation, indicating that CSP would not be significantly enhanced if the policy was not implemented. The improvement of CSP is affected by GCP, which passes the counterfactual test.

The CSP may be affected by other policies within the same time period after the implementation of the Green Credit Guidelines policy. For this reason, we sort out the relevant policies and regulations. The most representative ones are the ‘Green development’ and ‘Innovation development’ in the 13th Five-Year Plan issued in 2015, the ‘Opinions on innovation-driven development strategy’, and the new ‘Environmental protection law’ [24]. We use shortened time windows and introduce other policies for robustness tests to eliminate interference from these policies and regulations in this paper. In model (3), we shorten the time window of the sample to three years, which means that we re-perform the regression analysis using 376 observations from 2011–2013. The results show that the net effect coefficient of the policy is positively significant at the 1% level, which passes the robustness test of shortening the time window. The interaction term $Treated_{i}*Post_{15}$ of other government policies in 2015 is introduced in model (4) for robustness tests, and the model is shown below.

(4) $$Csp_{it}=\eta_{0}+\eta_{1}Treated\times Post12_{it}+\eta_{2}Treated_{i}\times Post_{15}+\eta_{3}Controls_{it}+Fixed\;effect+\varepsilon_{it}$$

where

i is the firm;

t is the year;

$\eta_{1}$ is the DID coefficient which measures the net effect of GCP on CSP in 2012;

$\eta_{2}$ is the DID coefficient which measures the net effect of other government policies on CSP in 2015;

Controls is the set of control variables;

Fixed effects is the two-way fixed effects;

$\varepsilon_{it}$ is the residual.

If the coefficient of the interaction term $Treated_{i}\times Post_{12}$ in model (4) is not significant, it indicates that the improvement of CSP is mainly affected by other policies. If the coefficient of the interaction term is significant, it proves that the conclusion in the baseline regression that GCP has a significant positive correlation with CSP is reliable. The regression results show that the coefficient of the interaction term $Treated_{i}\times Post_{12}$ is positively significant at the 1% level and that the coefficient of the interaction term $Treated_{i}\times Post_{15}$ is positively insignificant. Above all, the empirical test results of replacing the dependent variable, counterfactual, shortening the time window and introducing other policies are essentially consistent with the findings of the baseline regression, indicating that the findings of this paper are very reliable.

## 5. Expanded Research

### 5.1. Heterogeneity Test

Do differences in corporate internal characteristics and the external environment create heterogeneity in the relationship between GCP and CSP? To answer this question, we select the nature of property rights and green innovation level for the internal characteristics, and the regional characteristics and market concentration for the external environment, as the grouping basis. Table 6 shows that the impact of GCP on CSP is obviously limited by the internal characteristics of the firm and the external environment.

The results of grouping by the nature of property rights show that the relationship between GCP and CSP is only significantly positively at the 1% level in non-SOEs, and not in SOEs. This finding fully supports H2a. The main reason for this is that SOEs are in a special position as the mainstay of the national economy, with their funding and control coming from the government. The strong financial support from the government enables SOEs to face lower financing constraints. Stable R&D and investment budgets weaken the impact of environmental pollution on debt costs. Furthermore, the natural political connections of SOEs can amplify their green image; therefore, their sensitivity to GCP is lower. However, for non-SOEs, bank loans are their main source of financing. Commercial banks use environmental information as a criterion for granting loans. In the short term, this will have a significant negative impact on the financing ability of non-SOEs, but the forcing effect will encourage them to actively carry out technological innovation activities and establish a green image, thereby achieving green upgrading of the industrial structure.

The results of the regional grouping show that the relationship between GCP and CSP is only significantly positive at the 5% level in eastern enterprises, and is not significant in non-eastern enterprises. This finding fully supports H2b. The ecological civilization construction and sustainable development model not only stimulates development potential in the East, but also local governments are paying more attention to environmental issues, requiring companies to disclose social responsibility information and report substantive corporate social responsibility (CSR) activities [57]. These external objective environmental requirements are consistent with the purpose of GCP implementation. In contrast, the poor natural environment and lack of economic development potential in the central and western regions lead to companies preferring to pay fines rather than pollution control. Backwards environmental concepts and irrational industrial layouts result in no significant improvement in the financing environment in the central and western regions. In addition, the coordinated development of regional economies is a priority of the central government’s economic reforms. The implementation of a series of development strategies, such as western development, northeast revitalization and the rise of Central China, makes enterprises in non-eastern regions able to receive more financial subsidies, reducing their sensitivity to GCP.

The paper uses the Herfindahl–Hirschman Index (HHI) as a measure of industry concentration. The higher the market concentration is, the lower the competitive pressure within the industry. We refer to the US Department of Justice’s market structure classification criteria and classify industries with HHI <1000 as competitive, which means they are in a low level of market concentration group, and industries with HHI ≥1000 as oligopolistic, which means they are in a high level of market concentration group. The results of the market concentration grouping show that the relationship between GCP and CSP is only significantly positive at the 5% level for companies with low market concentration, and is not significant for companies with high market concentration. This finding fully supports H2c. The implementation of GCP stimulates companies with low market concentration to invest more in technological innovation to produce low-carbon, green products. The increased market share and improved external financing environment contribute to CSP. We use the number of green patents to measure the green innovation level of enterprises, and those with green patents during the year are classified as the high green innovation level group, while those without green patents during the year are classified as the low green innovation level group. The regression results for the grouping of the green innovation level show that the relationship between GCP and CSP is only significantly positive at the 5% level in high green innovation level companies, but is not significant in low green innovation level companies. The finding is consistent with the Porter effect theory, which verifies that the positive impact of GCP on CSP is due to the innovation compensation effect more than to the cost compliance effect. Enterprises should increase their investment in technological innovation and introduce green processes and technologies to improve their green innovation output.

### 5.2. Analysis of Mediating Effects

Baseline regressions indicate that the implementation of GCP significantly contributes to CSP. However, the impact mechanism needs to be further explored. As shown by the theoretical analysis in H3 above, GCP may have compensation effects through technological innovation, thereby improving CSP. To test whether TIL has a mediating effect on the relationship between GCP and CSP, this paper continues to construct the following models based on Equation (2):

(5) $$Rd_{it}=\nu_{0}+\nu_{1}Treated_{i}\times Post_{t}+\nu_{2}Controls_{it}+Fixed\;effect+\varepsilon_{it}$$

(6) $$Csp_{it}=\omega_{0}+\omega_{1}Treated_{i}\times Post_{t}+\omega_{2}Rd_{it}+\omega_{3}Controls_{it}+Fixed\;effect+\varepsilon_{it}$$

As shown by the theoretical analysis in H4 above, GCP may have a forcing effect through CRA and thus enhance CSP. To test whether the overall level of credit financing mediates the relationship between GCP and CSP, this paper continues to construct the following models based on Equation (2):

(7) $$Adf_{it}=\gamma_{0}+\gamma_{1}Treated_{i}\times Post_{t}+\gamma_{2}Controls_{it}+Fixed\;effect+\varepsilon_{it}$$

(8) $$Csp_{it}=\theta_{0}+\theta_{1}Treated_{i}\times Post_{t}+\theta_{2}Adf_{it}+\theta_{3}Controls_{it}+Fixed\;effect+\varepsilon_{it}$$

To test whether the level of long-term credit financing has a mediating effect on the relationship between GCP and CSP, this paper proceeds to construct the following models based on Equation (2).

(9) $$Ldf_{it}=\lambda_{0}+\lambda_{1}Treated_{i}\times Post_{t}+\lambda_{2}Controls_{it}+Fixed\;effect+\varepsilon_{it}$$

(10) $$Csp_{it}=\mu_{0}+\mu_{1}Treated_{i}\times Post_{t}+\mu_{2}Ldf_{it}+\mu_{3}Controls_{it}+Fixed\;effect+\varepsilon_{it}$$

where

i is the firm;

t is the year;

$\nu_{1}$ , $\omega_{1}$ *,* $\gamma_{1}$ , $\theta_{1}$ , $\lambda_{1}$ and $\mu_{1}$ are the DID coefficients;

$\nu_{2}$ , $\omega_{2}$ , $\gamma_{2}$ , $\theta_{2}$ , $\lambda_{2}$ and $\mu_{2}$ are the coefficients of the control variables;

Controls is the set of control variables;

Fixed effects is the two-way fixed effects;

$\varepsilon_{it}$ is the residual.

To enhance the accuracy of our findings, we adopt the bootstrap method to test the mediation effects of CRA and TIL. The algorithm follows the process procedure developed by Igartua and Hayes [58]. If the 95% confidence interval does not include 0, then there is a mediating effect; if the 95% confidence interval includes 0, then there is no mediating effect. In Table 7, model (1) and model (2) test whether TIL mediates the relationship between GCP and CSP. Model (1) shows that GCP has a significant positive relationship with TIL at the 1% level, but model (2) shows that the impact of TIL on CSP is not significant. Therefore, the causal stepwise regression method is not able to identify the mediating effect of TIL. We use the bootstrap method, and the results show that the 95% confidence interval for the coefficient on the interaction term $\nu_{1}\times \omega_{2}$ does not include 0, indicating that GCP improves CSP through TIL. This finding fully supports H3.

Model (3) and model (4) test whether the overall level of credit financing has a mediating effect on the relationship between GCP and CSP. Model (3) shows that GCP does not have a significant relationship with the overall level of credit financing, model (4) shows that the overall level of credit financing also does not have a significant effect on CSP. The bootstrap sampling test shows that the 95% confidence interval of the coefficient on the interaction term $\lambda_{1}\times \mu_{2}$ does not include 0, indicating that GCP improves CSP through the overall level of credit financing. Model (5) and model (6) test whether long-term credit financing has a mediating effect on the relationship between GCP and CSP. Model (5) shows that GCP has a negative relationship with long-term credit financing but is not significant. Model (6) shows that long-term credit financing also does not have a significant effect on CSP. The bootstrap sampling test shows that the 95% confidence interval of the coefficient on the interaction term $\gamma_{1}\times \theta_{2}$ does not include 0, indicating that GCP improves CSP through long-term credit financing. In summary, these findings fully support H4.

To summarize, the results of this paper are as follows: When using the DID model to study the economic consequences of policy, the problem of sample selection bias may easily arise. PSM is the best way to address this issue. Therefore, this paper explores the impact mechanism of GCP on CSP by constructing a PSM-DID model. The SMD values for most covariates decreased significantly after matching, and none of their absolute values exceeded 0.2, indicating that the matching effect is effective. The result of the causal effect test shows that the coefficient of the treatment group is significant at the 1% level ( Effect Size=0.048,p<0.01 ), and there is a causal effect between GCP and CSP. The results of the baseline regression show that GCP has a significant positive relationship with CSP at the 1% level ( $\alpha_{1}=0.043,p<0.01$ ), indicating that GCP improves CSP. The coefficient of the interaction term is not significant from 2010 to 2011 before policy implementation, indicating that there are parallel trends between the treatment and control groups after matching; only in the year 2012 policy implementation is the coefficient of the interaction term significant at the 5% level ( $\beta_{0}<0.05$ ), indicating that the effect of policy implementation diminishes over time. We use four methods to verify the reliability of the baseline regressions, including replacing the dependent variable, counterfactual testing, shortening the time window and introducing other policies, all of which pass the robustness test. In the heterogeneity analysis, we find that the impact of GCP on CSP is significantly limited by the internal characteristics of companies and the external environment. We further explore the impact mechanism of GCP on CSP and find that the 95% confidence intervals for the coefficients on the interaction terms $\nu_{1}\times \omega_{2},\;\;\lambda_{1}\times \mu_{2}$ and $\gamma_{1}\times \theta_{2}$ do not include 0 through a bootstrap sampling test, indicating that GCP enhances CSP through CRA and TIL.

## 6. Discussion

Stable monetary policy is a foundation for high-quality development of the real economy. The ‘Green Credit Guidelines’ policy is an effective measure of monetary policy to support the green upgrading of industrial structure and they are an important part of the deepening of financial reform and construction of a green financial system by the Chinese government. We find that GCP has a significant positive relationship with CSP. This finding is consistent with the research results of Jiang et al. [28]. The dynamic effect shows that GCP has a positive impact on CSP only in 2012 and 2013. The effect of policy implementation diminishes over time, and there is no long-term effect. This finding is consistent with the findings of Tian et al. [24] and Ren et al. [59]. The main reason is that Chinese green finance reform started late and has not developed comprehensive supporting measures for the implementation of GCP. Ambiguous policy details, unclear implementation standards, lack of environmental information, low quality of environmental information disclosure and government–business collusion are the main problems in the implementation of green credit policies [60]. Although the baseline regression results show that GCP significantly contributes to CSP, the validity of this finding requires us to test in depth. We performed robustness tests by replacing the dependent variable, performing counterfactual tests, shortening the time window and introducing other policies. The results of the robustness test by replacing the dependent variable show that GCP indeed significantly and positively affects CSP; the counterfactual test using a dummy time shows that the policy effect is not significant, indicating that the change of CSP comes from the implementation of GCP; however, since we set the sample time window as 2010–2015, it is possible that other government policies contributed to the change of CSP. To eliminate interference from other policies, we use shortened time windows and the introduction of other policies to test these findings. The results are generally consistent with the findings of the baseline regression, indicating that the findings of this paper are very reliable.

Heterogeneity analysis finds that there is heterogeneity in the impact of GCP on CSP in terms of the nature of property rights, green innovation level, regional characteristics and market concentration. The specific political background of SOEs gives them a stable R&D budget, a higher national mission and social responsibility. Whether GCP is implemented or not, they themselves are under pressure to make the green transformation. However, non-SOEs, whose debt financing mainly comes from commercial banks, have a greater sensitivity to GCP. To promote coordinative development among regions, the central government provides significantly higher financial subsidies to enterprises in the central and western regions than in the eastern regions. In addition, the traditional development model of polluting first and treating later, and the backwards concept of environmental protection, cannot be changed in a short period of time; therefore, GCP has limited impact on the central and western regions. Companies with low market concentration face higher competitive pressure and financing constraints. The implementation of GCP and high competitive pressures within the market are forcing companies with low market concentration to innovate and thereby gain first-mover advantages. The relationship between GCP and CSP is significantly positive for companies with high levels of green innovation, verifying that the positive impact of GCP can be attributed to the innovation compensation effect more than to the cost compliance effect.

Although the implementation of GCP increases the intensity of external financing constraints for enterprises in the short term, it also leads to the flow of social capital to green industries in the long term by adjusting CRA, achieving the goal of stimulating the green transformation of the industrial structure of HPEs. Moreover, as a type of environmental regulation, GCP indeed significantly increases TIL, which is consistent with the results of Wang et al. [61], thereby improving CSP through the innovation compensation effect. This paper not only verifies that CRA mediates the relationship between GCP and CSP, but also proves that a strong version of the ‘Porter effect’ is applicable to Chinese manufacturing companies [62]. 

## 7. Conclusions

### 7.1. Conclusions and Implications

This paper explores the impact mechanism of GCP on CSP using the 2012 ‘Green Credit Guidelines’ Policy as a quasi-natural experiment. The PSM method and DID method are combined to construct a PSM-DID model, which not only significantly reduces the sample selection bias but also improves the reliability of the results. The baseline regression shows that GCP has a significant positive impact on CSP, which supports H1. To explore whether the relationship between GCP and CSP is limited by the internal characteristics of companies and the external environment, this paper performs grouped regressions on four dimensions: nature of property rights, green innovation level, regional characteristics and market concentration. Heterogeneity tests fully support H2a, H2b and H2c. We use the bootstrap method to verify that CRA and TIL mediate the relationship between GCP and CSP, so H3 and H4 are also fully supported. 

This paper not only explores the economic consequences of GCP from the perspective of CRA and TIL, but also proposes the following set of meaningful insights for enterprises, government and financial institutions to achieve the green development goals of ‘carbon neutrality’ and ‘carbon peaking’:

Enterprises should optimize their capital allocation and improve their internal governance structure to enhance their sustainability. The implementation of GCP will inevitably directly lead to HPEs facing a higher intensity of financing constraints. Therefore, enterprises should focus on the environmental risks in their production and operation and optimize their capital allocation. In addition, the implementation of GCP will have a spillover effect, leading to a reduction in the availability of their commercial credit financing, which in turn will inhibit their technological innovation. Therefore, R&D activities should not rely excessively on bank borrowing and should also make full use of the capital market to introduce venture capital funds and seek financial support to achieve multiple sources of financing. The essential prerequisite for GCP to be effective is the behavioral choices of enterprises, and the level of environmental concern within enterprises and the educational background of executives may lead to different policy effects. Therefore, it is important to improve the internal governance of enterprises and raise managers’ awareness of environmental responsibility through green finance policies, thereby strengthening their understanding of the policies and establishing a good green image for the enterprises [16,28].

Financial institutions should improve the green credit auditing mechanism and create a favorable financing environment for green transformation enterprises. As the main implementer of GCP, financial institutions should improve green credit standards, evaluation systems and environmental information disclosure systems, strengthen the ability of GCP to internalize pollution costs, and accelerate the improvement of the overall level of environmental governance. In addition, financial institutions should strengthen information exchange with environmental departments, complete the information flow mechanism, and strengthen the guiding role of GCP to prevent enterprises from falling into a vicious cycle of financing difficulties and declining innovation levels. Referring to the ‘Equator Principles’ [63], we should comprehensively evaluate the contribution of enterprises in terms of environmental protection and social responsibility, implement lenient financing policies for green transformation enterprises to encourage their green development, and strengthen the development of green financial products such as green credit, green funds, green insurance, green stocks and bonds, and carbon finance to provide more financing options for enterprises’ technological innovation investment [62,64].

Government departments should improve the GCP system and increase policy implementation efforts. Government departments need to strengthen the supervision of financial institutions in the implementation of GCP and guide and implement risk compensation and incentive mechanisms for green loans to banks, carry out differential incentive measures and provide more financial subsidies to green transformation enterprises [65,66]. In addition, due to the stronger policy effects for non-SOEs, eastern regions and low-market-concentration enterprises, differential credit auditing standards should be developed by comprehensively considering the level of economic development and environmental governance pressures in different industries and regions. At present, China’s green credit system is not yet complete, and the long-term effects of the policy are not significant [24,57]. Therefore, combining GCP with other environmental regulation measures is conducive to developing an incentive mechanism for environmental social responsibility.

### 7.2. Limitations and Future Research

Certainly, this paper explores the economic consequences of GCP and enriches the research of CRA and green innovation theory, but there are still some limitations in the research process:

Commercial banks grant green credit based on corporate environmental information as the vetting criteria. Therefore, the quality of environmental information disclosure is an important influencing factor on companies’ access to credit resources, as well as an important way to build their green image and relieve financing constraints [45,54]. However, we did not study environmental information disclosure in our research process.

According to the SA index of financing constraints, company size is one of the most important factors affecting corporate financing [67]. This paper uses company size as a control variable to analyze its impact on CSP, and there are a large number of small and medium-sized enterprises (SMEs) in China, but we did not explore whether there is heterogeneity between GCP and CSP from the perspective of company size.

Debt and equity financing are the most important forms of corporate external financing. This paper only uses CRA as a mediating variable to explore whether it mediates the relationship between GCP and CSP. However, there is another situation in practice: when enterprises are blocked from green credit financing, they can turn to equity financing to achieve corporate green transformation. This issue should also be addressed in our research.

Summing up the above limitations, we will not only take the quality of environmental information disclosure, the impact of GCP on SMEs’ sustainable performance and the mediating effect of equity financing as future research directions, but we will also expand the scope of our research objects to explore the impact of GCP on sustainable performance from the perspective of commercial banks. In addition, in this paper we only use official government documents as a criterion to classify heavily polluting enterprises and non-heavily polluting enterprises. In a next step, we will use actual pollutant emissions of enterprises as a criterion to more accurately classify treatment and control groups. On the issue of measuring corporate sustainability performance, there is no uniform standard but we believe that it should include not only financial and environmental performance, but also social responsibility performance. Therefore, the environmental, social and governance (ESG) performance of enterprises can be used as a comprehensive indicator to measure CSP, and we will also further study the impact mechanism of different environmental regulation tools on ESG.

## Figures and Tables

**Table 1:** Variable definition and measurement.

Variable Category	Specific Indicators	Signs	Variable Description	Data Sources	Mean	Min	Max
Dependent variables	Financial performance	Roe	Ratio of net profit to equity	CNRDS Database Hexun.com Database	0.038	−7.220	0.823
	Environmental performance	Ep	Corporate environmental responsibility score		5.648	0.000	30.000
	Sustainability performance	Csp	Entropy of return on equity and environmental performance		0.193	0.000	0.901
Independent variables	Target of policy implementation	Treated	High polluters as 1, otherwise as 0	Wind Database	0.499	0.000	1.000
	Time of policy implementation	Post	After policy implementation as 1, otherwise as 0		0.667	0.000	1.000
	Net effect of policy implementation	Treated × Post	Policy target × time of policy implementation		0.338	0.000	1.000
Mediator	Total level of debt financing	Adf	Long- and short-term bank loans as a percentage of total assets		0.262	0.001	0.689
	Level of long-term debt financing	Ldf	Long-term bank loans as a percentage of total assets		0.088	0.000	0.479
	Level of technological innovation	Rd	Investment in research and development as a percentage of operating revenue		0.019	0.000	0.122
Control variables	Corporate size	Size	Natural logarithm of total assets	CSMAR Database	22.992	20.088	26.961
	Leverage ratio	Lev	Ratio of liabilities to assets		0.595	0.193	1.050
	Corporate growth	Growth	Operating revenue growth rate		0.217	−1.463	6.217
	Type of shareholding	State	State-owned enterprises as 1, otherwise as 0		0.685	0.000	1.000
	Ratio of independent directors	Idr	Ratio of independent directors to total board members		0.377	0.200	0.667
	Financial background of directors	Fbd	Financial background as 1, otherwise as 0		0.663	0.000	1.000
	Economic development level	Gdp	Natural logarithm of gross regional product	NBS Database	4.320	3.059	4.874
	Market development level	Mai	Market index	CMI Database	8.178	3.359	11.113

**Table 2:** Results of the balance test after propensity score matching.

Variables	Unmatched Cohort				Matched Cohort			
	Mean		*t*-Test		Mean		*t*-Test	
	Treated (*n* = 611)	Controls (*n* = 613)	*t*-Value	*p*-Value	Treated (*n* = 610)	Controls (*n* = 154)	*t*-Value	*p*-Value
*Size*	22.829	23.156	4.675	<0.001 ***	23.155	23.136	0.185	0.853
*Lev*	0.577	0.613	4.531	<0.001 ***	0.613	0.599	1.009	0.313
*Growth*	0.332	0.102	−2.116	0.034 **	0.103	0.116	−0.327	0.743
*State*	0.647	0.697	0.884	0.376	0.697	0.682	0.358	0.720
*Idr*	0.126	0.144	0.044	0.964	0.377	0.376	0.236	0.813
*Fbd*	0.684	0.643	−1.492	0.135	0.644	0.643	0.032	0.974
*Gdp*	4.333	4.307	−1.415	0.157	4.308	4.260	1.642	0.101
*Mai*	8.350	8.006	−3.149	0.001 ***	8.013	7.786	1.354	0.176

Note: **, *** Significant at 5% and 1% confidence levels, respectively.

**Table 3:** Test for causal effects after matching.

Effect	Est.	S.e.	z	*p* > |z|	[95% Conf. int.]	
ATE	0.049	0.012	3.976	0.000 ***	0.025	0.073
ATC	0.054	0.012	4.517	0.000 ***	0.031	0.078
ATT	0.048	0.012	3.381	0.000 ***	0.023	0.072

Note: *** Significant at 1% confidence levels, respectively.

**Table 4:** Main effects of GCP on CSP.

Dep. Variable	Model (1)	Model (2)	Model (3)
	*Csp*		
$Treated_{i}\times Post_{12}$	0.021 **		0.043 ***
	(2.12)		(2.64)
*Size*		−0.007	−0.011
		(−0.31)	(−0.46)
*Lev*		−0.042	−0.050
		(−0.62)	(−0.75)
*Growth*		0.008	0.006
		(0.64)	(0.47)
*State*		0.118 **	0.121 **
		(2.41)	(2.48)
*Idr*		0.099	0.094
		(0.70)	(0.67)
*Fbd*		0.026 *	0.024 *
		(1.78)	(1.66)
*Gdp*		0.134	−0.054
		(1.33)	(−0.44)
*Mai*		0.014	−0.011
		(−0.88)	(−0.75)
*Intercept*	0.583 ***	0.018	1.044 *
	(84.89)	(0.43)	(1.93)
*Effects*	Time	Time	Time
	Entity	Entity	Entity
No. Observations	764	764	764
R-Squared	0.008	0.020	0.032

Note: *, **, *** Significant at 10%, 5% and 1% confidence levels, respectively, with t-stats in parentheses.

**Table 5:** Results of robustness tests.

Dep. Variable	Model (1)	Model (2)	Model (3)	Model (4)
	*Csp_new*	*Csp*	*Csp*	*Csp*
$Treated_{i}\times Post_{10}$		−0.012		
		(−0.17)		
$Treated_{i}\times Post_{12}$	0.042 ***		0.066 ***	0.043 ***
	(2.65)		(2.87)	(2.62)
$Treated_{i}\times Post_{15}$				0.002
				(0.12)
*Control variables*	Yes	Yes	Yes	Yes
*Intercept*	1.032 *	0.193	3.614 **	1.063 *
	(1.94)	(0.44)	(2.32)	(1.88)
*Effects*	Time	Time	Time	Time
	Entity	Entity	Entity	Entity
No. Observations	764	764	376	764
R-Squared	0.034	0.020	0.069	0.032

Note: *, **, *** Significant at 10%, 5% and 1% confidence levels, respectively, with t-stats in parentheses.

**Table 6:** Results of the heterogeneity test.

Classifications	Property Rights Nature		Regional Characteristics		Concentration Ratio		Green Innovation Level	
	SOEs	Non-SOEs	East	Non-East	Low	High	Low	High
Dep. Variable	*Csp*							
$Treated_{i}\times Post_{12}$	0.017	0.062 ***	0.053 **	0.029	0.071 **	0.030	0.050	0.045 **
	(0.57)	(3.18)	(2.52)	(1.127)	(2.33)	(1.50)	(1.39)	(2.36)
*Control variables*	Yes	Yes	Yes	Yes	Yes	Yes	Yes	Yes
*Intercept*	0.327	1.388 ***	0.987	1.234	2.382 **	0.616	1.175	1.338 **
	(0.32)	(2.11)	(1.52)	(1.20)	(2.05)	(0.97)	(0.92)	(2.11)
*Effects*	Time	Time	Time	Time	Time	Time	Time	Time
	Entity	Entity	Entity	Entity	Entity	Entity	Entity	Entity
Observations	530	234	462	302	245	519	540	224
R-squared	0.033	0.093	0.057	0.034	0.111	0.026	0.004	0.044

Note: **, *** Significant at 5% and 1% confidence levels, respectively, with t-stats in parentheses.

**Table 7:** The mediating effect of credit resource allocation and technological innovation level.

Dep. Variable	Model (1)	Model (2)	Model (3)	Model (4)	Model (5)	Model (6)
	*Rd*	*Csp*	*Adf*	*Csp*	*Ldf*	*Csp*
$Treated_{i}\times Post_{12}$	7.195	0.051 ***	0.005	0.043 ***	−0.006	0.043 ***
	(9.23)	(2.92)	(0.56)	(2.63)	(−0.78)	(2.64)
*Adf*				0.010		
				(0.13)		
*Ldf*						0.023
						(0.24)
*Rd*		−0.001				
		(−1.26)				
*Control variables*	Yes	Yes	Yes	Yes	Yes	Yes
*Intercept*	−27.283	1.013 *	0.779 ***	1.036 *	0.581 **	1.031 *
	(−1.09)	(1.88)	(2.81)	(1.90)	(2.43)	(1.90)
*Effects*	Time	Time	Time	Time	Time	Time
	Entity	Entity	Entity	Entity	Entity	Entity
No. Observations	764	764	764	764	764	764
R-Squared	0.383	0.034	0.294	0.032	0.133	0.032
Bootstrap test	$\nu_{1}$ $\mathrm{is}\;\mathrm{significant},\;\omega_{2}$ is not significant, so Bootstrap test is necessary		$\gamma_{1}$ $\mathrm{and}\;\theta_{2}$ are not significant, so Bootstrap test is necessary		$\lambda_{1}$ $\mathrm{and}\;\mu_{2}$ are not significant, so Bootstrap test is necessary	
95% Confidence intervals	[−0.0176, −0.0014]		[0.0011, 0.0109]		[−0.0047, −0.0000]	
Mediation effect	Significant		Significant		Significant	

Note: *, **, *** Significant at 10%, 5% and 1% confidence levels, respectively, with t-stats in parentheses.

## Data Availability

Not applicable.

## Appendix A

**Table A1:** Industry Classification of Treatment and Control Groups.

Classification	Industries
Treatment group	Wine, beverage and refined tea manufacturing; textile industry; textile clothing and apparel industry; leather, fur, feather and feather products and footwear industry; paper and paper products industry; printing and recording media reproduction industry; petroleum processing, coking and nuclear fuel processing industry; chemical raw materials and chemical products manufacturing industry; pharmaceutical manufacturing industry; chemical fiber manufacturing industry; rubber and plastic products industry; non-metallic mineral products industry; ferrous metal smelting and calendering processing industry; non-ferrous metal smelting and rolling processing industry; metal products industry
Control group	Agriculture and food processing industry; food manufacturing industry; tobacco manufacturing industry; wood processing and wood, bamboo, rattan, palm and grass products industry; furniture manufacturing industry; cultural, educational, industrial, aesthetic, sporting and recreational goods manufacturing industry; general equipment manufacturing industry; special equipment manufacturing industry; automobile manufacturing industry; railway, ship, aerospace and other transportation equipment manufacturing industry; electrical machinery and equipment manufacturing industry; computer, communication and other electronic equipment manufacturing; instrumentation manufacturing; other manufacturing; comprehensive utilization of waste resources manufacturing; machinery and equipment repair manufacturing
